# Transcranial stimulation over right inferior frontal gyrus increases the weight given to private information during sequential decision-making

**DOI:** 10.1093/scan/nsy106

**Published:** 2018-11-27

**Authors:** Xiaofei Niu, Jianbiao Li, Glenn J Browne, Dahui Li, Qian Cao, Xiaoli Liu, Guangrong Wang, Pengcheng Wang

**Affiliations:** 1 Reinhard Selten Laboratory, China Academy of Corporate Governance, Business School, Nankai University, Tianjin, China; 2 Department of Economic and Management, Nankai University Binhai College, Tianjin, China; 3 School of Economics, Shandong University, Shandong, China; 4 Rawls College of Business, Texas Tech University, Lubbock, TX, USA; 5 Labovitz School of Business and Economics, University of Minnesota Duluth, Duluth, MN, USA; 6 Neural Decision Science Laboratory, Weifang University, Weifang, China; 7 International Business School, Tianjin University of Finance and Economics, Tianjin, China

**Keywords:** inferior frontal gyrus, weight of evidence, tDCS, sequential decision-making, information cascades

## Abstract

Decision makers often follow other similarly situated people in making decisions, creating a sequential decision-making context. Although rational behavior is often to make the same choice as previous decision makers, which can result in an information cascade, people may assign inappropriately higher weight to their own private information and discount public information about predecessors’ choices. Recent findings suggest that overweighting private information may be associated with increased activities in the inferior frontal gyrus (IFG). In the present study, we employed transcranial direct current stimulation (tDCS) and developed a computational model to examine the causal relationship between right IFG (rIFG) and overweighting private information. Specifically, we applied three types of tDCS over rIFG while participants were completing a sequential decision-making task. Our results showed that anodal stimulation significantly increased the weight given to private information and decreased the response time in making a decision when private information conflicted with public information, but cathodal stimulation did not have such impacts. Importantly, the effect of anodal stimulation was significant in some conditions when information conflict or task difficulty reached a threshold that might trigger cognitive control-related processes. Our findings revealed the important role of rIFG in trade-off between considering private and public information during sequential decision-making.

## Introduction

Individual decision makers are often faced with choices for which previous similarly situated decision makers have already made selections. If the choices of the previous decision makers are known, the new decision makers have two types of information—their own private information (which could consist of data, previous experiences, opinions, etc.) and the knowledge of the choices of the previous decision makers. Private information is nearly always incomplete and imperfect; such information will have some degree of validity and the decision maker must choose whether to rely on that information. Public information in terms of choices made by previous decision makers is assumed to be known and fully valid. In such situations, a rational decision maker will combine the public and private information using Bayes’ rule and will often make the same choice as previous decision makers even if the choice conflicts with what his or her private information suggests. When numerous sequential decision makers rationally follow previous decision makers and disregard their own private signals, the resulting sequence is known as an ‘information cascade’ (Banerjee, 1992; Bikhchandani *et al.*,^ 1992^). Information cascades have been observed in a variety of settings, such as technology adoption (Walden and Browne, 2009), movie ratings (Lee *et al.*,  2015) and revolutionary regime transitions (Ellis and Fender, 2011).

However, the literature concerning information cascades has found that there exist systematic deviations from the Bayesian Nash Equilibrium (BNE), which prescribes how a rational individual should condition his or her decision based on predecessors’ actions (Huck and Oechssler, 2000; Nöth and Weber, 2003; Kübler and Weizsäcker, 2004; Goeree *et al.*, 2007). These findings demonstrate that an individual may assign higher weight to his or her own private information relative to the publicly observable information from the decisions made by others. In fact, overweighting private information can lead to fewer cascades than predicted by BNE (Weizsäcker, 2010; see also, Walden and Browne, 2009). These results have important implications for theories of rational choice, suggesting that rational choice models often are not descriptive of human behavior (Simon, 1996). For this reason, investigating neural mechanisms to improve our understanding of overweighting private information is useful and can provide important insights into information cascades, weighting of evidence and decision-making more generally.

A recent functional magnetic resonance imaging (fMRI) study provided some initial insights into the neural mechanism to explain how an individual updates his or her private information as compared with public information in information cascades situations (Huber *et al.*, 2015). This study showed that the more a participant overweights private information, the higher the activation in the inferior frontal gyrus (IFG). Moreover, an individual’s tendency to overweight his or her private signal was associated with his or her degree of overconfidence (Nöth and Weber, 2003). Overconfident participants exhibited a tendency to allocate their initial attention to private signals (Innocenti *et al.*, 2010), indicating an attentional preference for these individuals of private over public information. Li *et al.*(2018) found in an event-related potential (ERP) study that the percentage of choices consistent with private information was correlated with frontal N200, which plays a key role in cognitive control (Folstein and Van Petten, 2008). Attention and cognitive control were found to be represented in the right IFG (rIFG) (Aron *et al.*, 2004; Hampshire *et al.*, 2010). Additionally, patients who had attention deficit hyperactivity disorder were able to improve interference control with anodal transcranial direct current stimulation (tDCS) over rIFG (Breitling *et al.*, 2016). The interference effect was significantly decreased after anodal tDCS over rIFG in an imitation-inhibition task (Hogeveen *et al.*, 2015).

These findings suggest that the activity of rIFG might be causally involved in overweighting private information during sequential decision-making. However, there is no experimental evidence so far, to the best of our knowledge, to support this speculation. In addition, prior findings of overweighting private information in the neuroscience literature have been based solely on fMRI or ERP (Huber *et al.*, 2015; Li *et al.*, 2018) and are not sufficient to reveal causal relationships between brain activity and human behavior. Establishing a causal relationship between rIFG and the overweighting of private information is crucial for our understanding of the neural mechanism that causes people to make non-rational choices in sequential decision-making situations.

To formally test the causal relationship, we conducted an experiment to study whether tDCS, a method of non-invasive stimulation of the human brain by means of weak currents, over rIFG affected overweighting of private information. In addition, we developed an information heteroweighting model (IHM) to describe the weight given to private information.

In the experiment, participants performed a sequential decision-making task adapted from the information cascades experiment of Anderson and Holt (1997). While participants were performing the task, we applied anodal, cathodal or sham stimulation over rIFG. Anodal and cathodal tDCSs are known to increase or decrease the resting potential, which leads to an increase or decrease of neural excitability in the targeted regions, while sham tDCS, which mimics the peripheral effects (i.e. tactile sensations), does not affect any neural processing (Nitsche *et al.*^2008^). Huber *et al.*(2015) found that people who tend to overweight private as compared with public information show increased activity in the IFG. Li *et al.*(2018) also found overweighting private information was correlated with control-related N200, which may stem from the rIFG region (Aron *et al.*, 2004; Hogeveen *et al.*, 2015; Breitling *et al.*, 2016). We therefore expected that, compared with sham stimulation, anodal stimulation over rIFG would increase rIFG activity (and possibly other connected areas) so that a decision maker would increase the weight given to private information, whereas cathodal stimulation might have the opposite effect.

## Materials and methods

### Participants

Ninety-eight healthy students from Nankai University volunteered to participate in this study [mean age, 22.3 years; standard error of mean (SEM), 0.19 years; range, 19–28 years; 31 men]. Each participant was given a 5 Chinese yuan ($0.76 US) participation fee and also received a variable amount of money at the end of the experiment based on his or her performance during the task. The university’s institutional review board approved all the experimental procedures and protocols.

Participants were randomly assigned to one of three stimulation groups: anodal (*n* = 34; 11 men), cathodal (*n* = 32; 12 men) or sham (*n* = 32; 8 men). We excluded two female participants from the anodal group because one participant reported discomfort with the stimulation and another failed to report answers within the response time (RT) limit in a majority of the trials. Overall, 96 participants successfully performed the task in the experiment.

All experimental sessions were conducted in a group room at the Reinhard Selten Laboratory. The group room was laid out with several enclosed cubicles, each of which was equipped with a computer that was connected to a local area network. All the computers had the same hardware and software configuration. This setting was designed to conduct anonymous and randomized experiments.

### Experimental tasks and procedure

Our sequential decision-making task was adapted from the information cascade experiment designed by Anderson and Holt (1997). In our study, a participant was asked to draw a conditionally independent private signal and predict which of two equally likely events had happened in the presence of known predictions made by prior participants. The events were denoted as *A* box and *B* box, and the signal was either *a* ball or *b* ball. Each of the two boxes (*A* and *B*) contained three balls (*a* or *b*), with the *A* box including two *a* balls and one *b* ball and the *B* box including one *a* ball and two *b* balls. In a total of 12 experimental trials, a participant received public information (i.e. the box designated but not the ball drawn) presented in an accumulated random order about the decisions made by predecessors.

At the beginning of a trial, one of the two boxes was randomly assigned to all participants from which to draw a ball and participants were then asked to predict from which box the ball was drawn. Predictions made by seven predecessors, representing public information, were then shown to the participant sequentially. The seven predecessors were not physically present in the experiment but had made their choices sequentially in a prior session and could observe each other’s choices. These people agreed that their choices could be used in subsequent sessions. A similar design was reported in Ruff *et al.*(2013). We arranged the seven people in a random order and indexed them as P1–P7. The participant in the experimental session then acted as P8 and drew a ball from the box assigned to all the predecessors in the trial. The participant was informed that P1–P7 also received their own conditionally independent private signals by drawing a ball from the same box. At the end of each trial, P8 made a prediction about which box the ball was drawn from after observing his or her own private signal (*a* ball or *b* ball) and the seven predecessors’ public information (*A* box or *B* box).

**Fig. 1:**
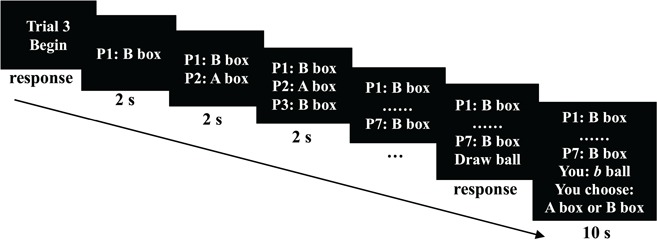
Time course of a single trial. At the beginning of each trial, a participant was presented with the current trial number followed by choices made by P1–P7. Once a participant pressed the ‘Begin’ button, P1’s choice was displayed on the screen. After 2 s, P2’s choice was displayed and so on. The seven predecessors’ choices were displayed sequentially, and the interval between displaying every predecessor’s choice was fixed at 2 s. After P7’s choice was displayed, P8 drew a ball by pressing the ‘Draw ball’ button. P8 then had 10 s to make a decision after observing his or her private signal.

The procedure of a single trial is depicted in Figure 1. In each trial, the predictions or choices made by P1–P7 were displayed at the center of the computer screen in a sequential order with an interval of 2 s between 2 predictions. The displays were cumulative in nature. Specifically, the choice made by P1 was first displayed. After 2 s, P2’s choice was presented together with that of P1. Then, the choices made by P3–P7 were displayed after every 2 s in a sequential order, while the choices made by the predecessors were still shown in the computer screen. After all the seven predecessors’ predictions were displayed, P8 pressed the ‘Draw ball’ button to receive his or her private signal. Once P8 received his or her private signal (*a* or *b*), he or she was given 10 s to designate from which box (*A* or *B*) he or she drew the ball. We recorded the prediction made by P8 and the RT for making the prediction. We programmed the experiment in z-Tree (Fischbacher, 2007).

Following a similar design (Huber *et al.*, 2015), we manipulated P8’s draw in two conditions (congruent *vs* incongruent) so that P8’s private signal was congruent with a majority of the seven predecessors’ choices in six trials and incongruent in the other six trials. We also followed Frydman and Krajbich (2017) and created a variable called Net Public Information for *P_i_* as ${\mathrm{NPI}}_i={(-1)}^{1+{1}_{a,i}}\times \sum \nolimits_{N=1}^{i-1}({1}_{A,N}-{1}_{B,N})$, *i* ϵ [1,7]. The first term, (${(-1)}^{1+{1}_{a,i}}$), is 1 if *P_i_* received private signal *a* and −1 if otherwise. The second term, ($\sum \nolimits_{N=1}^{i-1}({1}_{A,N}-{1}_{B,N})$), is the difference between the number of observed ‘*A*’ choices (or boxes) and those of ‘*B*’ choices. NPI*_i_* > 0 indicates that *P_i_* received a congruent private signal, while NPI*_i_* < 0 indicates an incongruent private signal. NPI had six distinct values in our study, which were 1, 3 and 5 in the congruent condition and −1, −3 and −5 in the incongruent condition.

We created a design matrix (2 × 3 × 2 = 12 trials; Table 1) by using all combinations of condition (incongruent *vs* congruent), absolute NPI (the difference between the number of ‘*A*’ choice and ‘*B*’ choice; 5 *vs* 3 *vs* 1) and P8’s private signal (ball *a vs b*).

**Table 1:** Stimuli in the main experiment

Condition	Absolute NPI	P1–P7’s choices (box)	P8’s private signal (ball)
Incongruent (NPI < 0)	5	*A-A-B-A-A-A-A*	*b*
		*B-B-A-B-B-B-B*	*a*
	3	*A-A-B-B-A-A-A*	*b*
		*B-B-A-A-B-B-B*	*a*
	1	*B-A-B-B-A-A-A*	*b*
		*A-B-A-A-B-B-B*	*a*
Congruent (NPI *>* 0)	5	*A-B-A-A-A-A-A*	*a*
		*B-A-B-B-B-B-B*	*b*
	3	*B-A-A-B-A-A-A*	*a*
		*A-B-B-A-B-B-B*	*b*
	1	*B-A-A-B-B-A-A*	*a*
		*A-B-B-A-A-B-B*	*b*

Compared with the design of short sequences of decisions in Huber *et al.*(2015), we report the neural correlates of overweighting private information from long sequences of decisions in sequential decision-making situations. In particular, a participant such as P8 would weigh the evidence from predecessors’ choices as well as his or her private draw. This weight of the evidence design makes it possible to rigorously examine the effects of tDCS over rIFG on overweighting private information in the different conditions of NPI. NPI measures the degree of information conflict between public and private signals. The trade-off between these two types of information underlies the tendency to start an information cascade (Walden and Browne, 2009; Weizsäcker, 2010).

In the experiment design of Huber *et al.*(2015), a participant was the third decision maker and the proportions were two-thirds or four-fifths that the ball (*a* or *b*) matched the label of the box (*A* or *B*). After receiving his or her private signal, a participant could update his or her belief, which corresponded to six different posterior probabilities. However, the NPI value was only 2 or −2. Thus, Huber *et al.*(2015) examined the neural activity of information cascades only for these levels of uncertainty and did not provide any neural evidence of whether the degree of information conflict moderated the overweighting of private information, which would lead to fewer cascades than predicted by BNE.

In our experimental design, we explore the differing weights given to private and public information. Importantly, we employ tDCS to examine the casual relationship between overweighting private information and rIFG for the eighth decision maker using various NPI values.

### Transcranial direct current stimulation

In the experiment, we applied tDCS in a double-blind sham-controlled setting by means of a battery-driven stimulator (Neuro Conn, Germany) in which weak currents were used to modulate regional neural excitability by increasing (anodal) or decreasing (cathodal) the resting membrane potential depending on the position and polarity of the electrode. In the present study, we applied anodal, cathodal and sham tDCS over an IFG region that was found to be activated in a prior fMRI study (Huber *et al.*, 2015). In line with previous tDCS studies that focused on IFG (Holland *et al.*, 2011; Hogeveen *et al.*, 2015), we centered the stimulation electrode on electrode site FC6, according to the standard 10–10 system that corresponds to rIFG. We used an electroencephalograph (EEG) cap to determine the electrode position for each participant, with the reference electrode posterior to the left mastoid (Breitling *et al.*, 2016). This reference electrode position minimizes its influence on other cortical areas that might be relevant to the top-down control of behaviors (e.g. other prefrontal regions). We applied tDCS using a set of standard electrodes fixed by rubber straps (5 × 7 cm; current density, 0.029 mA/cm^2^). These standard electrodes were chosen over customized focal electrodes because we planned to ensure that the large electrode covered all neural rIFG regions.

tDCS was applied for 20 min with 1 mA current strength for the anodal group and the cathodal group. The arrangement of the stimulation electrode was identical in all three groups except that the stimulator was turned off after 30 s for the sham group. In all groups, the current was applied with a 15 s ramp up and down.

Prior to the experiment, we randomly assigned each participant to a cubicle. While waiting for the installation of the experiment equipment, a participant filled out one questionnaire that included basic demographic information (e.g. age, gender and major) and another questionnaire that measured a person’s trust and respect, reciprocity, belief in fairness of others and norm of honesty based on the World Values Survey. After the installation of the experimental equipment, each participant received 5 min of tDCS to ensure stable stimulation effects (Nitsche *et al.*, 2008). During this stage, we explained the experimental instructions to the participant again and conducted three training trials.

The experiment was composed of 12 trials presented in a random order. At the end of the experimental task, the true box (*A* or *B*) from which a participant drew his or her private signal in each trial was revealed to the participant. Participants who predicted correctly in a trial received 4 Chinese yuan ($0.30 US) and 0 otherwise. The average pay-off for all participants over all trials was 40 Chinese yuan ($6.08) (range, $4.31–$7.16; s.d., $1.05).

In addition, we also asked each participant to fill out a questionnaire that measured the participant’s conformity tendency and personality traits while the tDCS stimulation was still ongoing. The conformity tendency was measured by asking the participant to report what choice P3 would make (*A* or *B* box) if P3 received an *a* ball after observing that both P1 and P2 chose the *B* box. The questions about personality traits included the cognitive reflection test (Frederick, 2005), degree of overconfidence (Ren and Croson, 2013), risk-taking attitudes (Holt and Laury, 2002) and loss aversion (Gächter *et al.*, 2007; Rau, 2014). Finally, participants indicated how much they perceived the stimulation to affect their behavior (using a 5-point Likert scale).

## A computational model and model fitting

To investigate further a decision maker’s weights given to private information and public information, we developed a computational model to describe how a decision maker updates his or her beliefs during sequential decision-making.

### Bayesian Nash Equilibrium

We use BNE to describe how a rational decision maker updates his or her beliefs in a sequential process, following Anderson and Holt (1997). We posit that P1, whose only information is his or her own private signal, will predict *A* box if he or she receives an *a* ball and *B* box if a *b* ball is received. Thus, P1 reveals his or her private signal (e.g. *a* ball) as public information (e.g. *A* box). If P2 receives a private signal (e.g. *a* ball) that matches P1’s choice (e.g. *A* box), P2 will reveal his or her private signal as public information by stating the *A* box also. If P2 receives a private signal (e.g. *b* ball) that does not match P1’s choice (e.g. *A* box), this will result in a posterior probability of one-half because the prior probability is one-half and the sample is balanced. Thus, P2 will likely state that he or she drew from the *B* box since his or her private signal was *b*. If P3 observes both P1 and P2 choosing the same box (e.g. *A*), and P3 receives a *b* ball, P3 will respond to an inferred sample of *a* balls on the first two draws and the *b* ball on his or her own draw. Let *n* be the number of relevant signals *a,* and *m* the number of relevant signals *b*. Bayes’ rule can be used to calculate the posterior probability of event *A*:

(1) \begin{equation*} \Pr \left(A\left|n,m\right.\right)=\frac{\Pr \left(n,m\left|A\right.\right)\Pr (A)}{\Pr \left(n,m\left|A\right.\right)\Pr (A)+\Pr \left(n,m\left|B\right.\right)\Pr (B)} \end{equation*}

In the example above, the posterior probability of *A* is greater than one-half and P3 should choose the *A* box in line with the public signal, disregarding his or her private signal. This generates an information cascade, with players *i* = 4, …, 8 adopting the same logic and selecting *A* box regardless of their own private signals. In sum, according to the prediction of BNE, the rational behavior for all subsequent decision makers is to choose the same event regardless of their private signals.

In our experiment, a participant could update his or her beliefs after receiving a private signal, which should correspond to six posterior probabilities in the different NPI values. Specifically, the posterior probability is 0.0588 (NPI = −5), 0.2 (NPI = −3) and 0.5 (NPI = −1) in the incongruent trials, and 0.8 (NPI = 1), 0.9412 (NPI = 3) and 0.9846 (NPI = 5) in the congruent trials. It may not be easy for participants to calculate the Bayes’ posterior probabilities of equation (1). But participants can simply count the numbers of observed ‘*A*’ choices (or boxes) and those of ‘*B*’ choices to approximate optimal decision-making in this situation.

### Information heteroweighting model

Despite the rationality assumptions noted above, previous studies using a similar experimental design have shown systematic deviations from predictions based on BNE (see Weizsäcker, 2010, for a review). Here we develop an IHM to identify how people weight private information in their decision-making. Bayes’ theorem assumes that people weight evidence according to normative principles. In our IHM, we measure how people actually weight private and public information, and *β* represents the weight given to private information. This model is consistent with previous experimental results that people overweight private information (e.g. Nöth and Weber, 2003; Goeree *et al.*, 2007). Our model attempts to describe how people actually weight information rather than making the normative assumption. Ours is thus a descriptive model that captures potential deviations from the normative ideal.

Suppose that *P_i_* receives an *a* ball, which denotes his or her private signal. The number of predecessors’ choices of *A* box is *n* − 1 and *B* box is *m*. That is, the number of relevant public signals *a* is *n* − 1, and the number of relevant public signals *b* is *m*. Let *β* be the weight *P_i_* gives to his or her private signal *a*. *γ_a_* and *γ_b_* refer to the weights *P_i_* gives to each relevant public signal *a* and *b*. Assume that *P_i_* has no bias toward public information and weights each relevant public signal *a* and *b* with 1 (i.e. *γ_a_* = *γ_b_* = 1),$\sum \nolimits_1^{n-1}{\gamma}_a=n-1$ and $\sum \nolimits_1^m{\gamma}_b=m$. Then, given *P_i_*’s private signal *a* and predecessors’ choices, the posterior probability of event *A* is

(2) \begin{align*} {\displaystyle \begin{array}{l}\Pr \left(A\left|n,m\right.\right)=\frac{\Pr \left(n-1+a,m\left|A\right.\right)\Pr (A)}{\Pr \left(n-1+a,m\left|A\right.\right)\Pr (A)+\Pr \left(n-1+a,m\left|B\right.\right)\Pr (B)}\\ {}=\frac{{\left[\Pr \left(a\left|A\right.\right)\right]}^{\beta }{\left[\Pr \left(a\left|A\right.\right)\right]}^{n-1}{\left[\Pr \left(b\left|A\right.\right)\right]}^m\Pr (A)}{{\left[\Pr \left(a\left|A\right.\right)\right]}^{\beta }{\left[\Pr \left(a\left|A\right.\right)\right]}^{n-1}{\left[\Pr \left(b\left|A\right.\right)\right]}^m\Pr (A)+{\left[\Pr \left(a\left|B\right.\right)\right]}^{\beta }{\left[\Pr \left(a\left|B\right.\right)\right]}^{n-1}{\left[\Pr \left(b\left|B\right.\right)\right]}^m\Pr (B)}\end{array}} \end{align*}

In our experiment design, $\Pr (A)=\Pr (B)=\frac{1}{2}$, $\Pr (A|a.)=\Pr (B|b.)=\frac{2}{3}$ and $\Pr (A|b.)=\Pr (B|a.)=\frac{1}{3}$. So equation (2) can be simplified as

\begin{align*} \Pr \left(A\left|n,m\right.\right)&=\frac{{\left(\frac{2}{3}\right)}^{\beta}\times {\left(\frac{2}{3}\right)}^{n-1}\times {\left(\frac{1}{3}\right)}^m\times \left(\frac{1}{2}\right)}{{\left(\frac{2}{3}\right)}^{\beta}\!\times\! {\left(\frac{2}{3}\right)}^{n-1}\!\times\! {\left(\frac{1}{3}\right)}^m\!\times\! \left(\frac{1}{2}\right)+{\left(\frac{1}{3}\right)}^{\beta}\!\times\! {\left(\frac{1}{3}\right)}^{n-1}\!\times\! {\left(\frac{2}{3}\right)}^m\!\times\! \left(\frac{1}{2}\right)}\nonumber\\&=\frac{1}{1+{2}^{\left(m-n\right)-\left(\beta -1\right)}} \end{align*}

\begin{align*} \frac{d\Pr }{d\beta}&=-1\times {\left(1+{2}^{(m-n)-(\beta -1)}\right)}^{-2}\times {2}^{(m-n)-(\beta -1)}\times (-1)\nonumber\\&=\frac{2^{(m-n)-(\beta -1)}}{{\left(1+{2}^{(m-n)-(\beta -1)}\right)}^2}>0. \end{align*}

If a participant weights public and private information equally, i.e. *β* = 1, equation (2) is identical to equation (1). However, if a participant gives higher weight to private information, *β* > 1; otherwise, *β* < 1.

### Model fitting

We fitted the computational model that described how much weight each participant gave to private information (*β*) in each condition of NPI. The parameter *β* in each NPI was computed by putting a participant’s percentage of choices consistent with his or her own private signal to the left side of equation (3).

## Data analyses and results

We analyzed the choice made by a participant and RT as dependent variables in two mixed-design analyses of variance (ANOVAs) with four factors. Condition (incongruent *vs* congruent), absolute NPI (5 *vs* 3 *vs* 1) and P8’s private signal (*a vs b*) were within-subject factors, and stimulation (anodal *vs* sham *vs* cathodal) was a between-subject factor. Choice was coded as a dummy variable and was set to 1 if a participant made a choice consistent with his or her own private signal and 0 if otherwise. Significant main and interaction effects were further analyzed using Bonferroni-corrected *post hoc* tests. In addition, we performed ordinary least squares (OLS) regression analyses with robust standard errors clustered at the individual (participant) level. All reported *P*-values are two-tailed and corrected for multiple comparisons.

With choice as the dependent variable, we found that the main effects of condition (*F*_1,93_ = 448.802; *P* < 0.001; partial *η*^2^ = 0.828) and absolute NPI (*F*_1.80,167.77_ = 65.097; *P* < 0.001; partial *η*^2^ = 0.412) were significant. The interaction effect of condition × absolute NPI (*F*_1.84,170.94_ = 59.86; *P* < 0.001; partial *η*^2^ = 0.392) was also significant.

The significant interaction effect of condition × absolute NPI suggests that a participant’s choice was strongly influenced by the congruency between P8’s private signal and the seven public choices made by predecessors. The tendency for P8 to make a choice consistent with his or her private information only held for trials in the incongruent condition (NPI < 0: *F*_2,92_ = 57.703, *P* < 0.001, partial *η*^2^ = 0.556; NPI = −5 *vs* NPI = −1: *P* < 0.001; NPI = −3 *vs* NPI = −1: *P* < 0.001; NPI = −5 *vs* NPI = −3: *P* = 0.97). P8 was more likely to make a choice in line with his or her private signal as NPI increased (NPI = −5, 19.791 ± 3.2%; NPI = −3, 22.917 ± 3.4%; NPI = −1, 63.021 ± 3.7%) in the incongruent condition. For the congruent condition (NPI > 0), almost all the participants (96%) made choices consistent with their private signals.

In addition, there was a significant main effect of P8’s private signal (*F*_1,93_ = 8.259; *P* = 0.005; partial *η*^2^ = 0.082), but the interaction effects between the private signal and other factors were not significant (all *P*s > 0.10). The surprising main effect of P8’s private signal suggests that P8 was more likely to make a choice in line with his or her private signal when receiving an *a* ball than a *b* ball (68.23 ± 1.51 *vs* 64.06 ± 1.48%). We return to this surprising result later.

The most important findings were the main effect of stimulation (*F*_2,93_ = 4.643; *P* = 0.012; partial *η*^2^ = 0.091) and the condition × stimulation interaction effect (*F*_2,93_ = 6.165; *P* = 0.003; partial *η*^2^ = 0.117). The simple effects analysis suggested that stimulation over rIFG did not change P8’s choice when P8 received a congruent signal (main effect of stimulation in the congruent condition: *F*_2,93_ = 1.068; *P* = 0.348; partial *η*^2^ = 0.022) but increased the number of choices consistent with private signal when the participant received an incongruent signal (main effect of stimulation in the incongruent condition: *F*_2,93_ = 5.995; *P* = 0.004; partial *η*^2^ = 0.114).

**Fig. 2:**
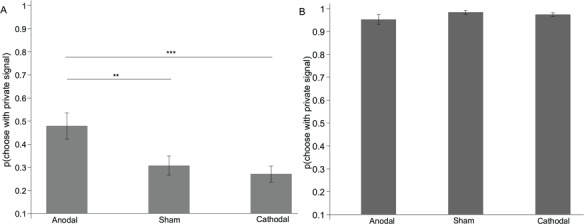
Impact of stimulation on the percentage of choices consistent with private signals. Anodal stimulation led to a higher percentage of choices consistent with private signals than both cathodal and sham stimulation in the incongruent condition **(A)**. There was no significant difference among the stimulation in the congruent condition **(B)**. Error bars indicate ±1 SEM. Asterisks indicate significance levels ^**^P < 0.05 and ^***^P < 0.01. (A) was for the incongruent condition; (B) was for the congruent condition.

Figure 2 shows the impact of stimulation on the percentage of choices consistent with the private signal. It shows that for the incongruent condition anodal rIFG stimulation resulted in a significantly higher percentage of choices in line with the private signal than both the sham stimulation (47.916 *vs* 30.729%; *P* = 0.025; Cohen’s *d* = 0.48) and the cathodal stimulation (47.916 *vs* 27.083%; *P* = 0.006; Cohen’s *d* = 0.64). The sham stimulation and cathodal stimulation did not differ (30.729 *vs* 27.083%; *P* = 0.687; Figure 2A). For the congruent condition, the anodal stimulation decreased the percentage of choices consistent with private signals more than the cathodal and sham stimulations (Figure 2B). However, this result was not significant (all *P*s > 0.60).

**Fig. 3:**
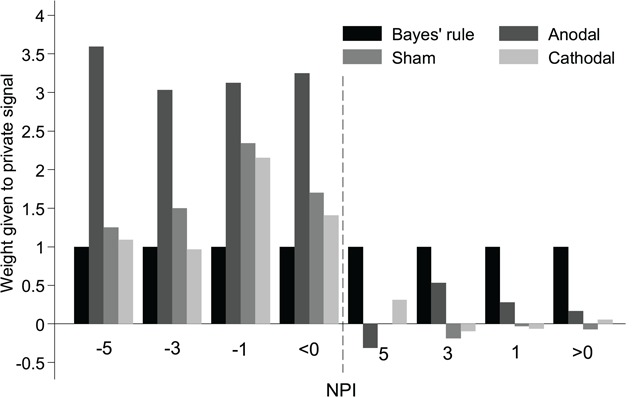
Model fits of the weight given to private information depend on NPI and rIFG stimulation. Anodal stimulation of the rIFG led to a higher weight given to private signals, as evident in the significantly higher *β* in the trials for NPI < 0.

Critically, model fits of the weight given to private information, as shown in Figure 3, indicated that participants deviated from BNE in all the stimulation groups. In particular, participants in the anodal group gave higher weight to private information in the incongruent condition. Further, the weight given to private information was significantly higher in the anodal group (*β* = 3.25 ± 0.505) than in the sham group (*β* = 1.698 ± 0.363; *P* = 0.015; Cohen’s *d* = 0.51) and the cathodal group (*β* = 1.406 ± 0.267; *P* = 0.002; Cohen’s *d* = 0.55), indicating anodal tDCS stimulation increased the weight given to private information. However, there were no significant differences among the three stimulation groups in the congruent condition (anodal, *β* = 0.167 ± 0.161; sham, *β* = −0.073 ± 0.044; cathodal, 0.052 ± 0.111; all *P*s > 0.15).

When testing RT as the dependent variable, a mixed-design ANOVA indicated that there were significant main effects of condition (*F*_1,93_ = 203.531; *P* < 0.001; partial *η*^2^ = 0.686) and absolute NPI (*F*_1.78,165.62_ = 24.884; *P* < 0.001; partial *η*^2^ = 0.211). The interaction effect of condition × absolute NPI was also significant (*F*_1.99,3.98_ = 6.484; *P* = 0.002; partial *η*^2^ = 0.065). *Post hoc* tests suggested that RT was significant between NPI = −5 (*M* = 1.979 ± 0.178 s) and NPI = −3 (*M* = 2.609 ± 0.212 s; *P* = 0.035), NPI = −5 and NPI = −1 (*M* = 3.661 ± 0.248 s; *P* < 0.001) and NPI = −3 and NPI = −1 (*P* = 0.001) in the incongruent condition. RT was also significant between NPI = 5 (*M* = 0.443 ± 0.080 s) and NPI = 3 (*M* = 0.880 ± 0.125 s; *P* = 0.002) and NPI = 5 and NPI = 1 (*M* = 1.182 ± 0.157 s; *P* < 0.001) in the congruent condition. RT significantly increased when NPI increased in the incongruent condition and decreased when *NPI* increased in the congruent condition. The highest RT was found when NPI was −1 (63.021% of participants chose consistent with their private signals). In addition, the main and interaction effects of P8’s private signal were insignificant (all *P*s > 0.10).

**Fig. 4:**
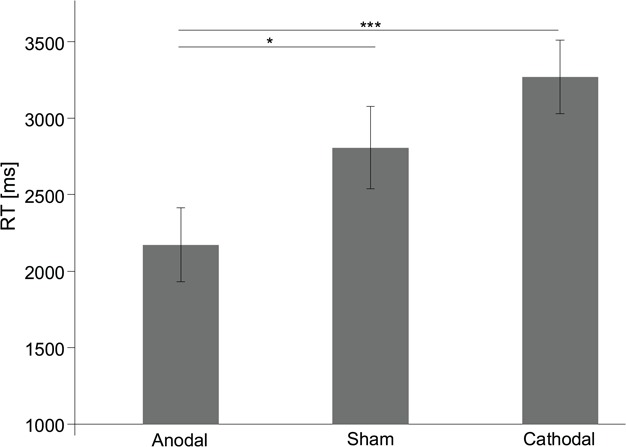
Impact of stimulation on the RT in the incongruent condition. Anodal stimulation led to a shorter RT than both cathodal and sham stimulations in the incongruent condition. Error bars indicate ±1 SEM. Asterisks indicate significance levels ^*^P < 0.10 and ^***^P < 0.01.

Importantly, we found a significant main effect of stimulation (*F*_2,93_ = 3.505; *P* = 0.034; partial *η*^2^ = 0.070) as well as a significant interaction effect of condition × stimulation (*F*_2,93_ = 3.833; *P* = 0.025; partial *η*^2^ = 0.076) on RT. The interaction effect revealed that stimulation had a significant effect on a participant’s RT in the incongruent condition (*F*_2,93_ = 4.814; *P* = 0.010; partial *η*^2^ = 0.094). Specifically, anodal stimulation resulted in shorter RT than both sham stimulation (2.172 *vs* 2.807 s; *P* = 0.062; Cohen’s *d* = 0.35) and cathodal stimulation (2.172 *vs* 3.270 s; *P* = 0.001; Cohen’s *d* = 0.47), while sham and cathodal stimulation did not differ (*P* = 0.143; Figure 4). In addition, stimulation did not significantly affect RT in the congruent condition (anodal, 0.745 ± 0.115 s; sham, 0.818 ± 0.169 s; cathodal, 0.943 ± 0.173 s; *P* > 0.10).

**Table 2:** Effect of tDCS on percentage of choices consistent with private signal in different NPI

NPI	Stimulation (*M* ± SEM)			*Post hoc* tests
	1: Anodal	2: Sham	3: Cathodal	
−5	0.359 ± 0.055	0.125 ± 0.055	0.109 ± 0.055	1 and 2^**^, 1–3^***^ and 2 and 3
−3	0.359 ± 0.060	0.188 ± 0.060	0.141 ± 0.060	1 and 2^*^, 1–3^**^ and 2 and 3
−1	0.719 ± 0.064	0.609 ± 0.064	0.563 ± 0.064	1 and 2, 1–3 and 2 and 3
5	0.969 ± 0.022	1 ± 0.022	0.969 ± 0.022	1 and 2, 1–3 and 2 and 3
3	0.938 ± 0.026	0.969 ± 0.026	0.984 ± 0.026	1 and 2, 1–3 and 2 and 3
1	0.953 ± 0.025	0.984 ± 0.025	0.969 ± 0.025	1 and 2, 1–3 and 2 and 3

^*^P < 0.10; ^**^P < 0.05; ^***^P < 0.01.

**Table 3:** Effect of tDCS on RT in different NPI

NPI	Stimulation [*M* ± SEM (seconds)]			*Post hoc tests*
	1: Anodal	2: Sham	3: Cathodal	
−5	1.765 ± 0.292	2 ± 0.308	2.171 ± 0.323	1 and 2, 1–3 and 2 and 3
−3	1.968 ± 0.319	2.812 ± 0.353	3.046 ± 0.421	1 and 2^*^, 1–3^**^ and 2 and 3
−1	2.781 ± 0.364	3.609 ± 0.446	4.593 ± 0.468	1 and 2^*^, 1–3^***^ and 2 and 3
5	0.484 ± 0.122	0.422 ± 0.121	0.422 ± 0.169	1 and 2, 1–3 and 2 and 3
3	0.937 ± 0.199	0.906 ± 0.238	0.796 ± 0.210	1 and 2, 1–3 and 2 and 3
1	0.813 ± 0.143	1.125 ± 0.270	1.609 ± 0.356	1 and 2, 1–3 and 2 and 3

^*^P < 0.10; ^**^P < 0.05; ^***^P < 0.01.

In addition, we did not find a significant three-way interaction effect of condition × absolute NPI × stimulation on the participant’s choice and RT (all *P*s > 0.10). However, Tables 2 and 3 show that anodal stimulation strongly affected the number of choices consistent with private signals and RT when NPI < 0. We therefore performed two additional ANOVAs with NPI (−5 *vs* −3 *vs* −1 *vs* 5 *vs* 3 *vs* 1) × P8’s private signal × stimulation as the factors.

The results confirmed that the impact of anodal stimulation depended on NPI (NPI × stimulation interaction: for choice, *F*_6.58,306.23_ = 2.774, *P* = 0.010, partial *η*^2^ = 0.056; for RT, *F*_7.22,335.64_ = 1.933, *P* = 0.062, partial *η*^2^ = 0.040). Specifically, anodal stimulation only increased the number of choices consistent with private signals when NPI = −3 (*F*_2,93_ = 3.721, *P* = 0.028, partial *η*^2^ = 0.074; anodal, 35.9 ± 6.0 *vs* sham, 18.8 ± 6.0%, *P* = 0.053, Cohen’s *d* = 0.46) and NPI = −5 (*F*_2,93_ = 6.497, *P* = 0.002, partial *η*^2^ = 0.123; anodal, 35.9 ± 5.5 *vs* sham, 12.5 ± 5.5%, *P* = 0.010, Cohen’s *d* = 0.67; Table 2) and decreased RT when NPI = −1 (*F*_2,93_ = 4.475, *P* = 0.014, partial *η*^2^ = 0.088; anodal, 2.781 ± 0.364 *vs* sham, 3.609 ± 0.446 s, *P* = 0.061, Cohen’s *d* = 0.36) and NPI = −3 (*F*_2,93_ = 2.383, *P* = 0.098, partial *η*^2^ = 0.049; anodal, 1.968 ± 0.319 *vs* sham, 2.812 ± 0.353 s, *P* = 0.073, Cohen’s *d* = 0.44; Table 3).

To further assess the effects of anodal and cathodal brain stimulations on participants’ number of choices consistent with their private signals and RT, we ran OLS regression analyses in which we defined ‘anodal’ and ‘cathodal’ as dummy variables. The variable ‘anodal’ was set to 1 if the individual received anodal stimulation or to 0 in all other cases. The variable ‘cathodal’ was set to 1 if the individual received cathodal stimulation or to 0 in all other cases. As shown in Supplementary data, Table S1, these analyses indicate that anodal tDCS increased the number of choices consistent with the private signal (coefficient, 0.172; *P* = 0.017) and decreased RT (coefficient, −0.635; *P* = 0.082) in the incongruent conditions but had no significant effect in the congruent condition. In the cathodal group, coefficients in the incongruent and congruent conditions were all insignificant (all *P*s > 0.10).

Finally, we ran four robustness checks to control the perception of tDCS and background variables (Robustness check I), cognitive complexity and conformity tendency (Robustness check II), overconfidence (Robustness check III) and risk-taking attitude and loss aversion (Robustness check IV; see the Supplementary data).

## Supplementary tDCS experiments

We also performed two additional tDCS experiments. The first tDCS experiment had 36 trials. This experiment tested the order effect to examine how much a participant relied on the anterior, middle or posterior public information to be consistent (or inconsistent) with his or her private signal. The second tDCS experiment had 24 trials in which a participant was the seventh person (P7) to make a choice. This experiment tested the effect of sequential order, examining whether our results were robust when we fixed participants’ position at P7.

### Supplementary tDCS experiment with 36 trials

In the first supplementary tDCS experiment, we utilized 36 trials for which a design matrix (2 × 3 × 3 × 2 = 36 trials; Table 4) was created by using all combinations of condition (incongruent *vs* congruent), absolute NPI (the difference between the number of ‘*A*’ choices and ‘*B*’ choices; 5 *vs* 3 *vs* 1), order of (in)consistent public information (order of public information consistent with the private signal in the incongruent condition and inconsistent with the private signal in the congruent condition; anterior *vs* middle *vs* posterior) and P8’s private signal (ball *a vs b*). All other aspects of the experimental design and procedure were identical to the main experiment. Thirty-six students (anodal, *n* = 18; sham, *n* = 18) participated in this experiment.

**Table 4:** Stimuli in the supplementary experiment with 36 trials

Condition	Absolute NPI	P1–P7’s choices (box)		P8’s private signal (ball)
Incongruent (NPI < 0)	5	Anterior	*A-B-A-A-A-A-A*	*b*
			*B-A-B-B-B-B-B*	*a*
		Middle	*A-A-B-A-A-A-A*	*b*
			*B-B-A-B-B-B-B*	*a*
		Posterior	*A-A-A-A-A-B-A*	*b*
			*B-B-B-B-B-A-B*	*a*
	3	Anterior	*B-A-A-B-A-A-A*	*b*
			*A-B-B-A-B-B-B*	*a*
		Middle	*A-A-B-B-A-A-A*	*b*
			*B-B-A-A-B-B-B*	*a*
		Posterior	*A-A-A-A-B-B-A*	*b*
			*B-B-B-B-A-A-B*	*a*
	1	Anterior	*B-A-B-B-A-A-A*	*b*
			*A-B-A-A-B-B-B*	*a*
		Middle	*B-A-A-B-B-A-A*	*b*
			*A-B-B-A-A-B-B*	*a*
		Posterior	*B-A-A-A-B-B-A*	*b*
			*A-B-B-B-A-A-B*	*a*
Congruent (NPI *>* 0)	5	Anterior	*A-B-A-A-A-A-A*	*a*
			*B-A-B-B-B-B-B*	*b*
		Middle	*A-A-B-A-A-A-A*	*a*
			*B-B-A-B-B-B-B*	*b*
		Posterior	*A-A-A-A-A-B-A*	*a*
			*B-B-B-B-B-A-B*	*b*
	3	Anterior	*B-A-A-B-A-A-A*	*a*
			*A-B-B-A-B-B-B*	*b*
		Middle	*A-A-B-B-A-A-A*	*a*
			*B-B-A-A-B-B-B*	*b*
		Posterior	*A-A-A-A-B-B-A*	*a*
			*B-B-B-B-A-A-B*	*b*
	1	Anterior	*B-A-B-B-A-A-A*	*a*
			*A-B-A-A-B-B-B*	*b*
		Middle	*B-A-A-B-B-A-A*	*a*
			*A-B-B-A-A-B-B*	*b*
		Posterior	*B-A-A-A-B-B-A*	*a*
			*A-B-B-B-A-A-B*	*b*

**Fig. 5:**
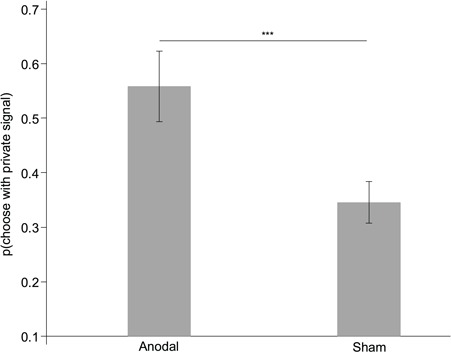
Impact of stimulation on the percentage of choices consistent with private signals in the incongruent condition for the supplementary tDCS experiment with 36 trials. Error bars indicate ±1 SEM. Asterisks indicate significance level ^***^P < 0.01.

Consistent with previous findings, when testing choice as the dependent variable we found a significant main effect of stimulation (*F*_1,34_ = 5.422; *P* = 0.026; partial *η*^2^ = 0.138) and an interaction effect of condition × stimulation (*F*_1,34_ = 9.039; *P* = 0.005; partial *η*^2^ = 0.210). For the incongruent condition, participants undergoing anodal tDCS had a higher percentage of choices consistent with the private signal than those undergoing sham tDCS (55.864 ± 6.454 *vs* 34.568 ± 3.799%; *P* = 0.007; Figure 5). But for the congruent condition anodal and sham, tDCS did not differ (95.444 ± 2.541 *vs* 99.074 ± 2.502%; *P* = 0.231). We also found that participants in the anodal group gave higher weight to private information in the incongruent condition (anodal, *β* = 3.909 ± 0.652; sham, *β* = 1.733 ± 0.323; *P* = 0.005), but in the congruent condition no significant difference was found between the anodal and sham stimulation groups (*P* = 0.421).

In addition, we found a significant main effect of order of (in)consistent public information (*F*_1.95,66.34_ = 3.882; *P* = 0.026; partial *η*^2^ = 0.102) as well as a marginal significant main effect of P8’s private signal (*F*_1,34_ = 3.114; *P* = 0.087; partial *η*^2^ = 0.084) on choice, but the interaction effects between order of (in) consistent public information (or P8’s private signal) and other factors were not significant (all *P*s > 0.10). Participants were more likely to make a choice in line with their private signal when the order of (in) consistent public information was in the posterior (74.074 ± 2.189%) than in the anterior (68.981 ± 2.312%; *P* = 0.025), while the middle (69.907 ± 1.805%) and the posterior (or anterior) did not differ (all *P*s > 0.10). The percentage of choices consistent with the private signal was higher when participants received an *a* ball than a *b* ball (72.07 ± 1.82 *vs* 69.91 ± 1.96%).

With RT as the dependent variable, there was a marginal interaction effect of condition × stimulation (*F*_1,34_ = 3.283; *P* = 0.079; partial *η*^2^ = 0.088). For the incongruent condition, RT in the anodal stimulation was shorter than that in the sham stimulation (1.336 ± 0.338 *vs* 2.114 ± 0.338 s; *P* = 0.052; Cohen’s *d* = 0.38). For the congruent condition, RT did not differ between the anodal and sham stimulations (0.448 ± 0.120 *vs* 0.509 ± 0.120 s; *P* = 0.605).

The results of two additional ANOVAs on choice and RT with NPI (−5 *vs* −3 *vs* −1 *vs* 5 *vs* 3 *vs* 1) × order of consistent or inconsistent choice × P8’s private signal × stimulation as the factors showed that there was a significant interaction effect of NPI × stimulation on choice (*F*_1.82,61.79_ = 5.705; *P* = 0.007; partial *η*^2^ = 0.144) as well as a marginal significant interaction effect of NPI × stimulation on RT (*F*_2.52,85.82_ = 2.815; *P* = 0.081; partial *η*^2^ = 0.060). Consistent with previous findings, anodal stimulation only increased the percentage of choices consistent with the private signal when NPI = −3 (anodal, 0.398 ± 0.078; sham, 0.194 ± 0.078; *P* = 0.013; Cohen’s *d* = 0.66) and NPI = −5 (anodal, 0.380 ± 0.065; sham, 0.194 ± 0.065; *P* = 0.052; Cohen’s *d* = 0.32) and decreased RT when NPI = −1 (anodal, 1.694 ± 0.459 s; sham, 2.852 ± 0.459 s; *P* = 0.021; Cohen’s *d* = 0.44) and NPI = −3 (anodal, 1.426 ± 0.404 s; sham, 2.213 ± 0.404 s; *P* = 0.061; Cohen’s *d* = 0.30).

### Supplementary tDCS experiment with participants in the seventh position

To test the effect of sequential order, we conducted a supplementary tDCS experiment in which a participant was the seventh person (P7) to make a choice. We created a design matrix (2 × 2 × 3 × 2 = 24 trials; Table 5) by using all combinations of condition (incongruent *vs* congruent), absolute NPI (the difference between the number of ‘*A*’ choice and ‘*B*’ choice; 4 *vs* 2), order of (in)consistent public information (order of public information consistent with the private signal in the incongruent condition or inconsistent with the private signal in the congruent condition; anterior *vs* middle *vs* posterior) and P7’s private signal (ball *a vs b*). All other aspects of the experimental design and procedure were identical to the main experiment. Thirty-one students (anodal, *n* = 15; sham, *n* = 16) participated in this experiment.

**Table 5:** Stimuli in the supplementary experiment with the seventh position

Condition	Absolute NPI	P1–P6’s choices (box)		P7’s private signal (ball)
Incongruent (NPI < 0)	4	Anterior	*A-B-A-A-A-A*	*b*
			*B-A-B-B-B-B*	*a*
		Middle	*A-A-B-A-A-A*	*b*
			*B-B-A-B-B-B*	*a*
		Posterior	*A-A-A-A-A-B*	*b*
			*B-B-B-B-B-A*	*a*
	2	Anterior	*B-B-A-A-A-A*	*b*
			*A-A-B-B-B-B*	*a*
		Middle	*A-A-B-B-A-A*	*b*
			*B-B-A-A-B-B*	*a*
		Posterior	*A-A-A-A-B-B*	*b*
			*B-B-B-B-A-A*	*a*
Congruent (NPI *>* 0)	4	Anterior	*A-B-A-A-A-A*	*a*
			*B-A-B-B-B-B*	*b*
		Middle	*A-A-B-A-A-A*	*a*
			*B-B-A-B-B-B*	*b*
		Posterior	*A-A-A-A-A-B*	*a*
			*B-B-B-B-B-A*	*b*
	2	Anterior	*B-B-A-A-A-A*	*a*
			*A-A-B-B-B-B*	*b*
		Middle	*A-A-B-B-A-A*	*a*
			*B-B-A-A-B-B*	*b*
		Posterior	*A-A-A-A-B-B*	*a*
			*B-B-B-B-A-A*	*b*

**Fig. 6:**
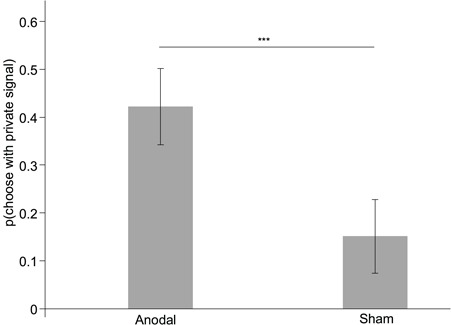
Impact of stimulation on the percentage of choices consistent with private signals in the incongruent condition for the supplementary tDCS experiment with participants in the seventh position. Error bars indicate ±1 SEM. Asterisks indicate significance level ^***^P < 0.01.

With choice as the dependent variable, we found a significant main effect of stimulation (*F*_1,29_ = 118.96; *P* < 0.001; partial *η*^2^ = 0.804) and an interaction effect of condition × stimulation (*F*_1,29_ = 7.043; *P* = 0.013; partial *η*^2^ = 0.195). For the incongruent condition, participants undergoing anodal tDCS had a higher percentage of choices consistent with their private signals than those undergoing the sham tDCS (42.222 ± 7.947 *vs* 15.104 ± 7.692%: *P* < 0.001; Figure 6). But for the congruent condition anodal and sham, tDCS did not differ (93.889 ± 2.361 *vs* 99.821 ± 2.286%: *P* = 0.214). We also found that participants in the anodal group gave higher weight to private information in the incongruent condition (anodal, *β* = 3.531 ± 0.534; sham, *β* = 1.973 ± 0.417; *P* = 0.011), but in the congruent condition no significant difference was found between the anodal and sham stimulation groups (*P* = 0.688).

In addition, we found a significant main effect of order of (in)consistent public information (*F*_1.86,53.99_ = 4.647; *P* = 0.016; partial *η*^2^ = 0.138) as well as a marginal significant main effect of P7’s private signal (*F*_1,29_ = 2.981; *P* = 0.091; partial *η*^2^ = 0.071) on choice, but the interaction effects between order of (in)consistent public information (or P7’s private signal) and other factors were not significant (all *P*s > 0.10). Participants were more likely to make a choice in line with the private signal when the order of (in)consistent public information was in the posterior (66.276 ± 3.129%) than in the anterior (62.239 ± 2.981%; *P* = 0.094) and in the middle (59.895 ± 2.514%; *P* = 0.024), but the latter two did not differ (*P* = 0.934). The percentage of choices consistent with the private signal was higher when participants received an *a* ball than a *b* ball (65.21 ± 2.81 *vs* 61.48 ± 2.76%).

With RT as the dependent variable, there was a marginal interaction effect of condition × stimulation (*F*_1,29_ = 3.455; *P* = 0.073; partial *η*^2^ = 0.106). For the incongruent condition, RT in the anodal stimulation was shorter than that in the sham stimulation (1.128 ± 0.378 *vs* 1.911 ± 0.338 s; *P* = 0.041; Cohen’s *d* = 0.51). For the congruent condition, RT did not differ between the anodal and sham stimulation (0.394 ± 0.093 *vs* 0.297 ± 0.090 s; *P* = 0.329).

The results of two additional ANOVAs on choice and RT with NPI (−4 *vs* −2 *vs* 4 *vs* 2) × order of consistent or inconsistent choice × P7’s private signal × stimulation as the factors showed that there was a significant interaction effect of NPI × stimulation on choice (*F*_1.27,36.91_ = 6.395; *P* = 0.011; partial *η*^2^ = 0.181) as well as a marginal significant interaction effect of NPI × stimulation on RT (*F*_1.41,40.77_ = 2.953; *P* = 0.080; partial *η*^2^ = 0.092). Anodal stimulation increased the percentage of choices consistent with the private signal when NPI = −4 (anodal, 0.400 ± 0.076; sham, 0.083 ± 0.073; *P* < 0.001; Cohen’s *d* = 0.61) and NPI = −2 (anodal, 0.444 ± 0.089; sham, 0.219 ± 0.086; *P* = 0.021; Cohen’s *d* = 0.53) and decreased RT when NPI = −4 (anodal, 0.856 ± 0.330 s; sham, 1.406 ± 0.320 s; *P* = 0.071; Cohen’s *d* = 0.33) and NPI = −2 (anodal, 1.400 ± 0.458 s; sham, 2.417 ± 0.444 s; *P* = 0.068; Cohen’s *d* = 0.28).

## Discussion

Information may be weighted in various ways by individuals in sequential decision-making situations. An information cascade occurs when it is optimal for someone who has observed the choices made by others before him or her to follow the behavior of the preceding individuals regardless of the content of his or her own private information. The probability that an information cascade starts depends primarily on how people weight their own private information as compared with public information. Several studies have found that people tend to overweight private information even when following others is the rational choice (e.g. Nöth and Weber, 2003; Goeree *et al.*, 2007). In the present experiment, our goal was to reveal the neural mechanism causing people to overweight private information. Specifically, we employed tDCS over rIFG to assess the role of the stimulated brain area in an individual’s tendency to overweight his or her private signal during a sequential decision-making task.

We found that anodal stimulation of rIFG significantly increased the weight given to private information and decreased RT in the incongruent condition (NPI < 0). This effect could not be attributed to individual differences in personality traits, such as conformity tendency and overconfidence, and it did not occur in the cathodal stimulation group. This finding supported the proposition in our IHM that people tend to give different weights to public and private information, which would have moderated the probability that a cascade occurs. We also found that a participant made more choices consistent with his or her own private signal when receiving an *a* signal than when receiving a *b* signal. Because the letter *a* is prior to the letter *b* in alphabetical order, it is possible that this order effect might have led to a preference for *a*. Thus, future studies might design an experiment to control for this potential effect. Furthermore, the results of our two additional tDCS experiments indicated that more weight was given to the private signal, and there was a shorter RT for anodal tDCS over rIFG in the incongruent condition when we controlled the order of P1–P7’s choices and when we fixed participants’ position to P7. This contributes to the robustness of our findings.

Our findings are consistent with the view that rIFG plays an important role in cognitive control (Aron *et al.*, 2004). The increased weight given to private information and decreased RT after anodal stimulation in the incongruent condition might have been due to a more general influence on cognitive control processes required for performing the task. Li *et al.*(2018) found that frontal N200 (250–340 ms) was more negative in the incongruent condition than in the congruent condition, and the percentage of choices consistent with private signals was correlated with the N200 amplitude. N200 was interpreted as a correlate of cognitive control and conflict processing (see Folstein and Van Petten, 2008, for a review). Previous ERP studies have found that non-matching stimuli elicit larger N200 than matching stimuli, and the detection of conflicts is central to eliciting N200 (Wang *et al.*, 2004). Moreover, comparing non-matching trials with matching trials produces rIFG activation (Hazeltine *et al.*, 2003). In our study, anodal stimulation over rIFG might have led to the higher weight given to private information only when NPI = −5 and NPI = −3, in which a participant encountered greater information conflict. However, if a participant had not received sufficient conflict between private and public information, choices made in the anodal group would have been similar to the other two stimulation groups.

Alternatively, assigning higher weight to a private signal after anodal stimulation of rIFG might have been explained by attentional control. Hampshire *et al.*(2010) suggested that rIFG plays a role in attentional control. Participants who assigned higher weight to private information allocated initial attention to private signals in 81% of the cases, while participants who followed BNE allocated gaze direction uniformly (Innocenti *et al.*, 2010). Anodal stimulation over rIFG might lead to higher weight given to private signals by allocating more attention to them while allocating less attention to public information. On the other hand, neuroimaging evidence has demonstrated the role of IFG in imitation learning (Buccino *et al.*, 2004). Anodal tDCS over rIFG showed a decrease in the interference effect in an imitation-inhibition task (Hogeveen *et al.*, 2015). Thus, anodal stimulation over rIFG might have ceased observational learning by enhancing the influence of private signals and reducing the influence of public signals. However, these two alternatives are not mutually exclusive and may work together to exert an effective overall influence.

Our findings also showed that anodal stimulation over rIFG decreased RT in incongruent trials, but this effect was only marginally significant when NPI = −3 and NPI = −1. According to sequential sampling models (SSMs; Ratcliff and Smith, 2004), shorter RT indicates easy decisions and longer RT indicates difficult decisions (Rubinstein, 2007). Our results supported the SSM prediction that the highest RT occurred when NPI = −1, while the shortest RT occurred when NPI = 5 (Table 3). Task difficulty might have explained why anodal stimulation over rIFG only decreased RT when NPI = −3 and NPI = −1. Our findings are consistent with recent tDCS studies (Jacobson *et al.*, 2011; Cunillera *et al.*, 2014; Stramaccia *et al.*, 2015), which have found that anodal stimulation over rIFG leads to significant reduction in the stop-signal RT (SSRT) in SS tasks. The SSRT reflects task difficulty and response inhibition level (Logan and Cowan, 1984).

Taken together, we found that the impact of anodal stimulation of rIFG on choices consistent with private signals and RT was specific to situations in which information conflict or task difficulty reached a threshold that might trigger cognitive control-related processing, presumably representing attentional control or attenuated observational learning. It is noteworthy that NPI = −3 showed a trade-off of information conflict and task difficulty. Anodal stimulation over rIFG both increased the weight given to private information and decreased RT when NPI = −3.

Although anodal stimulation over rIFG had a critical impact on the strength of overweighting private information, we posit that it is unlikely that rIFG is the only factor that drives this effect. Instead, rIFG stimulation might have altered the crosstalk of rIFG and other areas that are critical for decision-making, in particular the medial prefrontal cortex (mPFC) and ventromeadial PFC (vmPFC). mPFC has been shown to be involved in the valuation during value-based decision-making (Tom *et al.*, 2007) and in the integration of prior knowledge and likelihood information in calculating Bayesian posterior probabilities (Ting *et al.*, 2015). Campbell-Meiklejohn *et al.*(2017) used an urn task in which predecessors reported their choices as well as their associated confidence and showed that the value computed from private information and predecessors’ choices was indiscriminately represented across vmPFC. But the value derived from predecessors’ choices weighted by their confidence was represented with specificity for the ventromedial area 10, which may be explained by inferential and integrative processes. Similar to their urn task, in the present sequential decision-making experiment a participant should first observe predecessors’ choices and update his or her belief by forming an evaluation of the probability of the box being *A* or *B*. Our results provided evidence that rIFG might override mPFC and vmPFC activities and thus hinder an individual’s decision-making consistent with his or her private information when private information conflicted with public information. Thus, we conjecture that anodal stimulation of rIFG might moderate the interplay of prefrontal areas with areas involved in valuation and opinion formation so that an individual’s decision-making is biased toward cognitive control-related processes at the expense of ‘rational’ decision-making based on Bayes’ rule. That is, rIFG might interfere with Bayes inferential and integrative processes and mediate the weight given to each piece of information, which is accumulated by mPFC or vmPFC. This indicates that the moderating influence of rIFG on mPFC and vmPFC may not be ‘beneficial’. Further studies are needed to explore this relationship more carefully.

tDCS is a safe and non-invasive method that allows us to assess the role of cortical brain areas in cognitive processes such as decision-making. Based on previous fMRI results that identified IFG as a critical area for overweighting private information (Huber *et al.*, 2015), we chose an electrode position (FC6 in the standard EEG 10–10 system) that has been used in previous studies that targeted IFG (Holland *et al.*, 2011; Hogeveen *et al.*, 2015; Nobusako *et al.*, 2017). The reference electrode was positioned posterior to the left mastoid (Breitling *et al.*, 2016). It is important to note, however, that we did not exclusively stimulate rIFG, since the spatial resolution of tDCS is very limited. Using large electrodes (surface of 35 cm^2^) might produce wide spreading changes in cortical excitability, especially in the neighboring or other connected areas such as the dorsolateral prefrontal cortex (DLPFC). Previous studies have revealed the functions of DLPFC in cognitive control (MacDonald *et al.*, 2000) and self-control (Hare *et al.*, 2009). It is unlikely, however, that any unspecified tDCS effects that are independent of electrode position are responsible for our results because there was no significant improvement from the cathodal stimulation. In addition, small current density values predicted on brain and scalp model surface in Breitling *et al.*(2016) showed that shunting through low-resistive head tissues diminished largely when injecting through two large remote patch electrodes.

Finally, we did not find any significant effect of cathodal stimulation in spite of evidence of physiologically inhibitory influences by cathodal stimulation (Nitsche *et al.*, 2008). There are two plausible explanations for the lack of behavioral effects by cathodal stimulation. First, several studies proposed that the effect of cathodal stimulation may be task-dependent and induce less stable cognitive behavioral effects than anodal tDCS (e.g. Jacobson *et al.*, 2012). Second, it might reflect a floor effect, as the number of choices consistent with private signals in the sham condition was very small, especially when NPI = −3 and NPI = −5. This might have left little room for cathodal tDCS to decrease the number of choices.

To conclude, we have demonstrated in this study that anodal stimulation over rIFG increased the weight given to private information and decreased RT for the incongruent trials during sequential decision-making. This suggests that the stimulated brain area may play a critical role in reducing rational choice. Our findings of a malleable neural mechanism that influences the overweighting of private information are important to understand the cognitive process that causes individuals to violate rational decision-making in the form of information cascades. It is noteworthy that anodal tDCS over rIFG did not influence choices consistent with the private signal and RT in general but only occurred when information conflict or task difficulty reached a threshold that might trigger cognitive control-related processes. This is one of the most important findings in the present study.
